# Sunitinib induces coordinated mitochondrial, lysosomal, and endoplasmic reticulum disruption, leading to cellular collapse

**DOI:** 10.1117/1.BIOS.3.4.043102

**Published:** 2026-08-19

**Authors:** Aditya Yadav, Eugene Lee, Landen Liu, Jiajie Diao

**Affiliations:** University of Cincinnati College of Medicine, Department of Cancer Biology, Cincinnati, Ohio, United States

**Keywords:** sunitinib, cytotoxicity, structured illumination microscopy, organelle fragmentation, mitochondrial dynamics, lysosomal stress, endoplasmic reticulum disruption, intracellular localization, organelle connectivity index

## Abstract

**Significance:**

Understanding the subcellular mechanisms of sunitinib toxicity is critical for improving its therapeutic efficacy and minimizing adverse effects, as its intracellular mechanisms remain incompletely defined.

**Aim:**

We investigate the intracellular distribution and organelle-specific effects of sunitinib in HeLa cells using quantitative viability assays and structured illumination microscopy.

**Approach:**

Cell viability was assessed using CCK-8 assays. The intrinsic fluorescence of sunitinib allowed for live-cell imaging and colocalization analysis with organelle markers. Super-resolution imaging and quantitative morphometric analyses were used to evaluate mitochondrial, lysosomal, and endoplasmic reticulum integrity.

**Results:**

Sunitinib induced a dose-dependent reduction in cell viability. Imaging revealed preferential lysosomal accumulation. Mitochondria exhibited increased fragmentation and loss of hyperfused networks. Lysosomes appeared in reduced numbers while showing enlargement and decreased circularity, implying structural stress. ER networks displayed concentration-dependent fragmentation, as demonstrated by increased connectivity index values.

**Conclusions:**

Sunitinib induces coordinated structural and functional disruption across multiple organelles, highlighting organelle stress and loss of cellular homeostasis as key contributors to its cytotoxicity and intracellular pharmacodynamics.

Statement of Discovery This work provides new insight into the intracellular pharmacodynamics of sunitinib by demonstrating that its cytotoxicity is mediated not only through kinase inhibition but also through multi-organelle dysfunction and membrane-associated stress. In addition, our study highlights the value of super-resolution live-cell imaging for directly tracking drug localization and organelle-specific responses beyond the diffraction limit, offering a powerful framework for studying therapeutic molecules in complex cellular environments.

## 1 Introduction

Understanding how anticancer agents interact with cells at the subcellular level is crucial for advancing pharmacokinetic and pharmacodynamic design, improving therapeutic efficacy, and mitigating drug resistance.^1^^–^^3^ As intracellular trafficking and organelle interactions increasingly emerge as modulators of drug action, optical imaging technologies have become indispensable for directly visualizing these processes in real time. High-resolution fluorescence-based approaches now enable the precise mapping of drug molecule distribution, cellular uptake, organelle localization, and dynamic responses with unparalleled spatial and temporal detail, thereby bridging the gap between molecular pharmacology and cellular physiology.^4^^–^^7^

Sunitinib, a clinically approved multi-targeted tyrosine kinase inhibitor, is widely used for the treatment of metastatic renal cell carcinoma and other solid tumors.^8^^–^^10^ Although its therapeutic efficacy is traditionally attributed to the inhibition of receptor tyrosine kinases involved in tumor angiogenesis and proliferation, accumulating evidence suggests that its intracellular fate significantly influences both therapeutic and adverse responses as well.^11^^–^^15^ Recent studies have revealed that sunitinib both possesses intrinsic fluorescence and accumulates within acidic lysosomal compartments, contributing to cellular stress, altered signaling, and acquired drug resistance.^16^^,^^17^ Concurrently, there is growing recognition that sunitinib impacts multiple organellar systems, including mitochondria and the endoplasmic reticulum (ER), eliciting metabolic dysfunction and stress responses that extend beyond its canonical kinase-related activity.^18^^–^^20^ Such evidence suggests a broader, multi-organelle mechanism of action mediated by intracellular accumulation and organelle crosstalk, rather than solely receptor-level inhibition.

Mitochondria, lysosomes, the ER, and their crosstalk collectively govern cellular homeostasis, metabolism, and survival decisions under stress conditions.^21^^,^^22^ The disruption of these organelles often initiates cascades leading to apoptosis or autophagy, processes highly relevant to the outcome of cancer treatment. Notably, the weakly basic nature of sunitinib promotes lysosomal sequestration, which can both shield cells from cytotoxic effects and trigger lysosomal destabilization under specific conditions.^17^ Mitochondrial fragmentation and ER stress are increasingly implicated as amplifiers of sunitinib-induced cytotoxicity. Deciphering these multi-organelle interactions at the structural level is therefore essential to understanding the drug’s full intracellular pharmacodynamics and its mechanistic contributions to toxicity and resistance.

In this context, advanced optical imaging provides a powerful route to unravel complex drug-organelle interactions.^4^ High-resolution techniques such as structured illumination microscopy (SIM) permit direct visualization of organelle morphology, connectivity, and spatial co-localization, offering mechanistic insight beyond conventional biochemical or viability assays.^23^^–^^29^ For small, fluorescent molecules such as sunitinib, these methods enable the simultaneous assessment of drug localization and organelle integrity under controlled conditions, revealing how intracellular partitioning correlates with cellular stress and death pathways.

In the present study, we systematically investigated the effect of sunitinib on key organelles like mitochondria, lysosomes, and the ER in HeLa cells, combining quantitative cytotoxicity assays and super-resolution imaging. Our results unveil pronounced, concentration-dependent organelle disruption and fragmentation, highlighting lysosomal accumulation as a central cause of mitochondrial and ER dysfunction. By integrating imaging-based morphological analysis alongside quantitative metrics, this work both underscores the multifaceted nature of sunitinib toxicity and demonstrates how optical pharmacology can elucidate drug-induced cellular stress with sub-organelle precision. These insights build toward a deeper mechanistic understanding of intracellular drug behavior and inform future strategies for optimizing the efficacy and minimizing the toxicity of kinase inhibitors.

## 2 Result and Discussion

We first assessed the cytotoxic properties of the small-molecule drug sunitinib in HeLa cells using the Cell Counting Kit-8 (CCK-8) assay. HeLa cells are a reasonable choice here because they are a well-established, robust human cell line that supports reproducible subcellular drug studies and high-throughput experimental work. It should also be noted that, as an immortal cancer-derived line, HeLa cells may not fully capture the heterogeneity, microenvironment, or drug-response complexity of primary cancer cells or *in vivo* studies. Cells were treated with increasing concentrations of sunitinib (2.5 to 40  μM) for 24 h, followed by a 1 h incubation with the CCK-8 reagent to quantify cell viability based on optical density. As expected for a chemotherapeutic compound, sunitinib exhibited a pronounced dose-dependent cytotoxicity, with observable cell death occurring at concentrations as low as 2.5  μM (Fig. S1 in Supplementary Material). At 40  μM, the viability declined by more than 50%, confirming substantial toxicity at higher dosage levels.

To investigate the optical behavior of sunitinib, we characterized its intrinsic fluorescence properties using SIM. Fluorescence excitation at 488, 561, and 640 nm revealed that sunitinib emits strongly in the green channel upon 488 nm excitation (Fig. S2 in Supplementary Material), confirming intrinsic fluorescence suitable for direct visualization in live-cell imaging. Guided by this property, we explored the intracellular localization of sunitinib through co-staining experiments with organelle-specific dyes. HeLa cells were co-labeled with MitoTracker Deep Red (MTDR) for mitochondria and LysoTracker Red (LTR) for lysosomes. Then, they were treated with 20  μM sunitinib (Fig. S3 in Supplementary Material). SIM imaging demonstrated partial mitochondrial disruption after drug exposure, whereas colocalization analysis revealed a significantly higher Pearson’s correlation coefficient (PCC) between sunitinib and the lysosomal marker than between sunitinib and the mitochondrial marker (Fig. S4 in Supplementary Material). These findings indicate that sunitinib accumulates predominantly in lysosomes while simultaneously affecting mitochondrial organization.

**Fig. 1:**
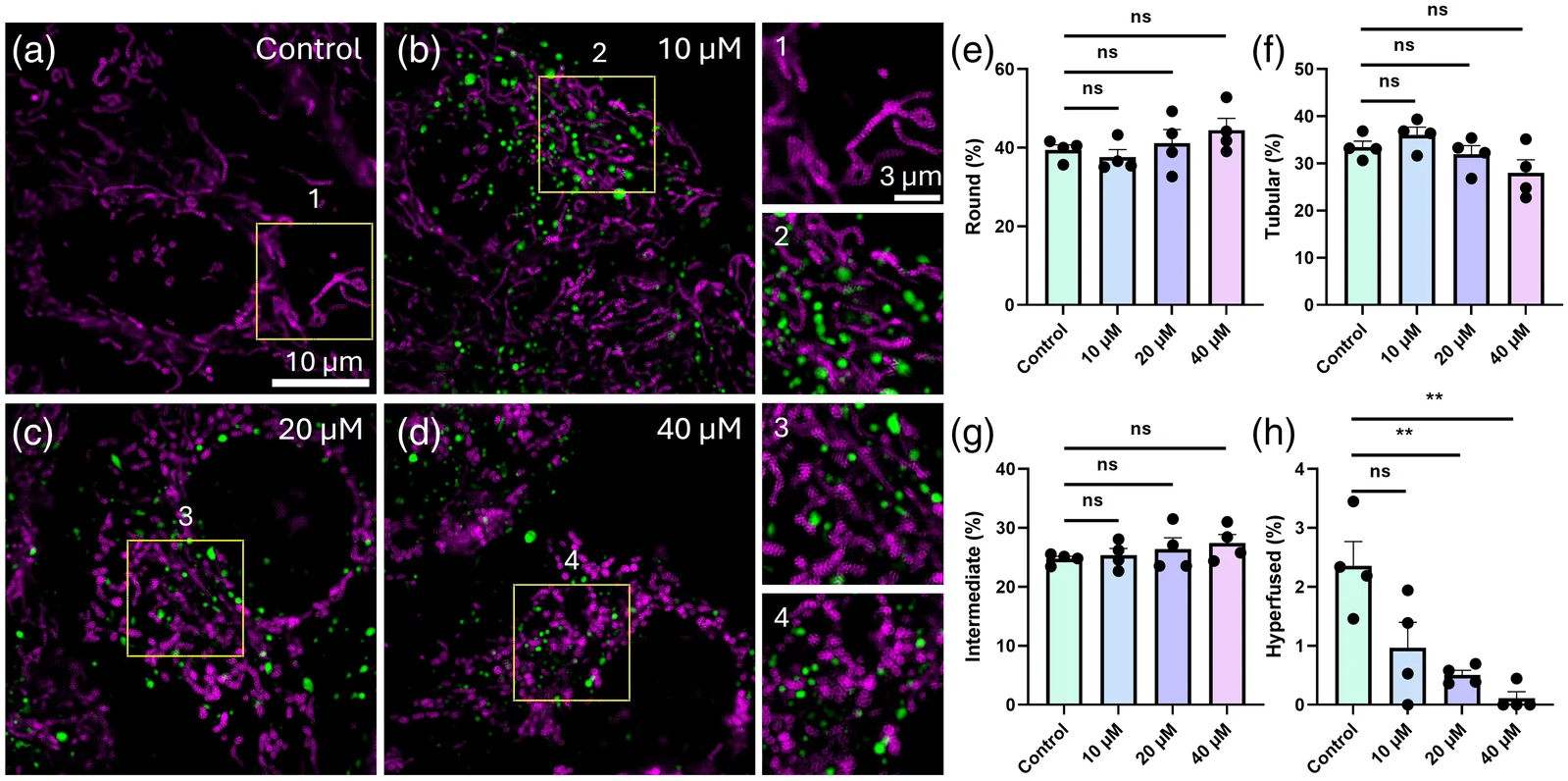
Effect of sunitinib on mitochondrial morphology in HeLa cells. (a) SIM image of untreated HeLa cells showing the characteristic reticular mitochondrial network. (b)–(d) SIM images following treatment with sunitinib at 10, 20, and 40  μM, respectively, depicting mitochondria (magenta) and sunitinib fluorescence (green). Increasing sunitinib concentration results in progressive disruption and fragmentation of the mitochondrial architecture. Zoomed-in views of selected regions of interest, outlined by yellow boxes in panels (1)–(4), highlight detailed structural alterations at higher magnification. (e)–(h) Quantitative distributions of mitochondrial morphology classes, including round, tubular, intermediate, and hyperfused, categorized according to aspect ratio (length/width) analysis. Data are presented as mean ± SEM (n=4). Statistical comparisons were performed using an unpaired t-test, assuming equal variance between groups; p<0.05 was considered statistically significant. *p<0.05, **p<0.01, ***p<0.001.

To evaluate the detailed morphological consequences of sunitinib exposure on mitochondria, HeLa cells co-stained with MTDR and sunitinib were analyzed at multiple concentrations (10 to 40  μM). SIM imaging revealed a progressive loss of mitochondrial integrity: the characteristic elongated, interconnected mitochondrial network at lower concentrations gave way to compact, fragmented, and swollen forms at higher doses [Figs. 1(a)–1(d)]. To quantify these morphological transitions, we calculated the mitochondrial length-to-width ratio (L/W) for each cell and categorized the structures as hyperfused (5.0≤L/W), tubular (2.0≤L/W<5.0), intermediate (1.5≤L/W<2.0), or round (1.0≤L/W<1.5) [Figs. 1(e)–1(h)]. Statistical analysis revealed that, although the proportions of round, intermediate, and tubular mitochondria remained largely unchanged across treatment conditions, the fraction of hyperfused mitochondria decreased sharply with increasing sunitinib concentration. The observed shift from elongated filaments to shorter fragments indicates a disruption of mitochondrial fusion-fission balance, potentially linked to altered activity of dynamic regulators such as MFN1/2 and DRP1.^30^^–^^32^ This structural decline indicates impaired mitochondrial integrity and function, suggesting that sunitinib-induced cytotoxicity involves direct damage to the organelle and disruption of energy metabolism. This damage may be driven by activation of mitochondrial fission pathways, which can also promote apoptotic signaling through the loss of membrane integrity and failure of cellular bioenergetics.

**Fig. 2:**
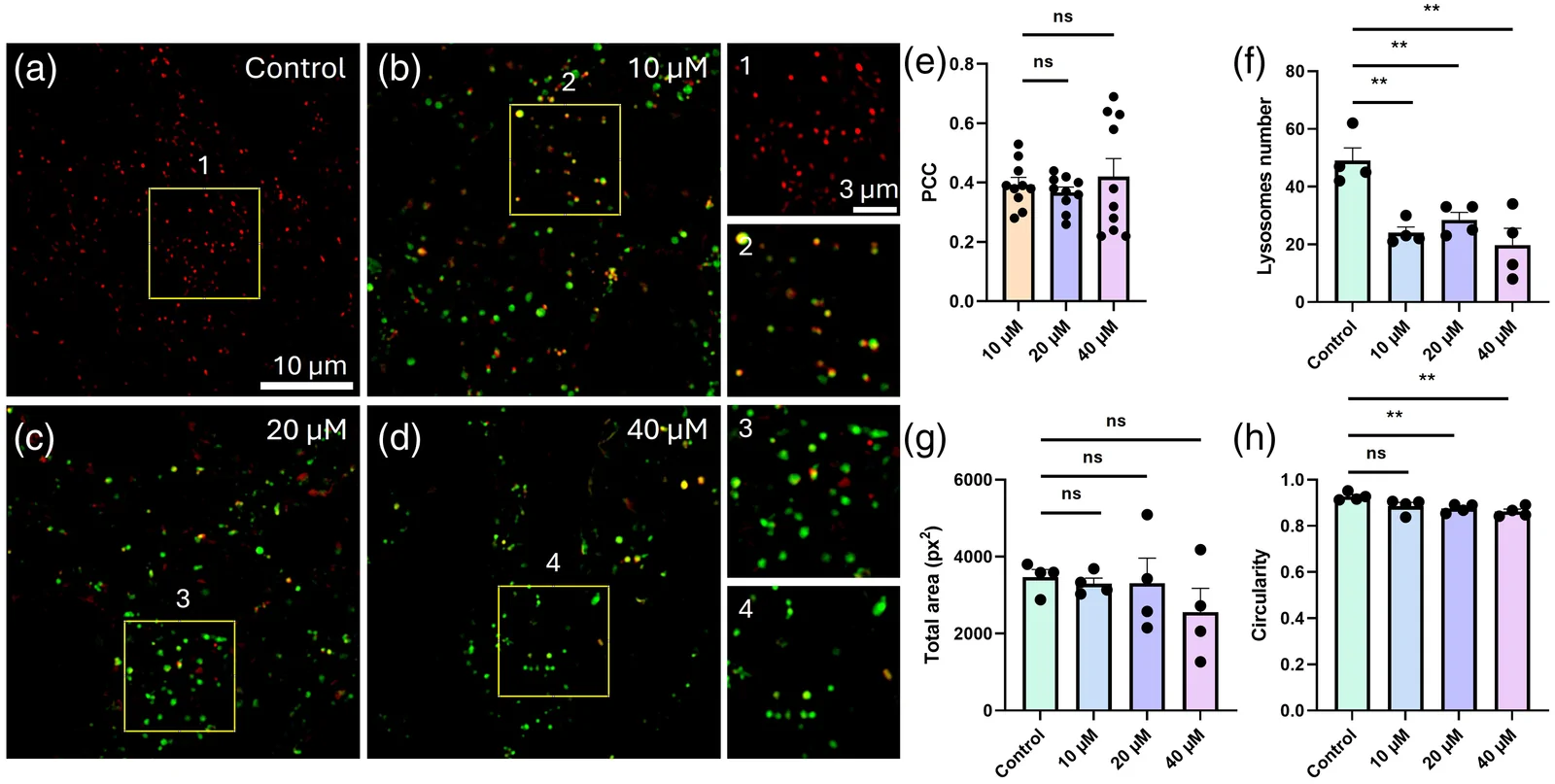
Effect of sunitinib on lysosomal morphology in HeLa cells. (a) SIM image of untreated HeLa cells showing normal lysosomal distribution and morphology. (b)–(d) SIM images of cells treated with sunitinib at 10, 20, and 40  μM, respectively, depicting lysosomes (red) and sunitinib fluorescence (green). Increasing drug concentration leads to progressive alteration in lysosomal morphology. Zoomed-in regions of interest, outlined by yellow boxes in panels (1)–(4), provide high-resolution views of structural changes. (e) PCC between the red and green channels indicate colocalization of lysosomes and sunitinib. (f) Quantification of lysosome numbers as a function of sunitinib concentration. (g) Total lysosomal area measured at varying drug concentrations. (h) Changes in lysosomal circularity with increasing sunitinib dosage. Data are presented as mean ± SEM (n=4). Statistical comparisons were performed using an unpaired t test assuming equal variance between groups; p<0.05 was considered statistically significant. *p<0.05, **p<0.01, ***p<0.001.

Given the preferential localization of sunitinib in lysosomes, we next examined its impact on lysosomal organization. HeLa cells were incubated with increasing drug concentrations (10 to 40  μM) and stained with LTR [Figs. 2(a)–2(d)]. Analysis of PCC values between sunitinib and LTR confirmed stable lysosomal accumulation across all concentrations [Fig. 2(e)], suggesting concentration-independent targeting. However, quantitative image analysis revealed a pronounced concentration-dependent decrease in the number of discrete lysosomal puncta per cell [Fig. 2(f)]. Interestingly, the total lysosomal area per cell remained relatively constant [Fig. 2(g)], implying that the loss in lysosome number may be offset by swelling or expansion of the remaining organelles. Such enlargement likely represents an adaptive response, where lysosomes increase in volume to sequester or degrade intracellular drug aggregates. Morphometric analysis further demonstrated a significant decrease in lysosomal circularity at higher sunitinib doses [Fig. 2(h)], indicating structural deformation and membrane destabilization.^33^ Collectively, these findings demonstrate that sunitinib accumulates within lysosomes and induces pronounced morphological alterations, which are indicative of lysosomal stress and dysregulated fusion dynamics. Such structural perturbations are consistent with compromised lysosomal function and may disrupt autophagic degradation pathways. Given the central role of lysosomes in autophagy, these alterations likely reflect impaired autophagic flux and activation of lysosome-dependent stress signaling mechanisms.

**Fig. 3:**
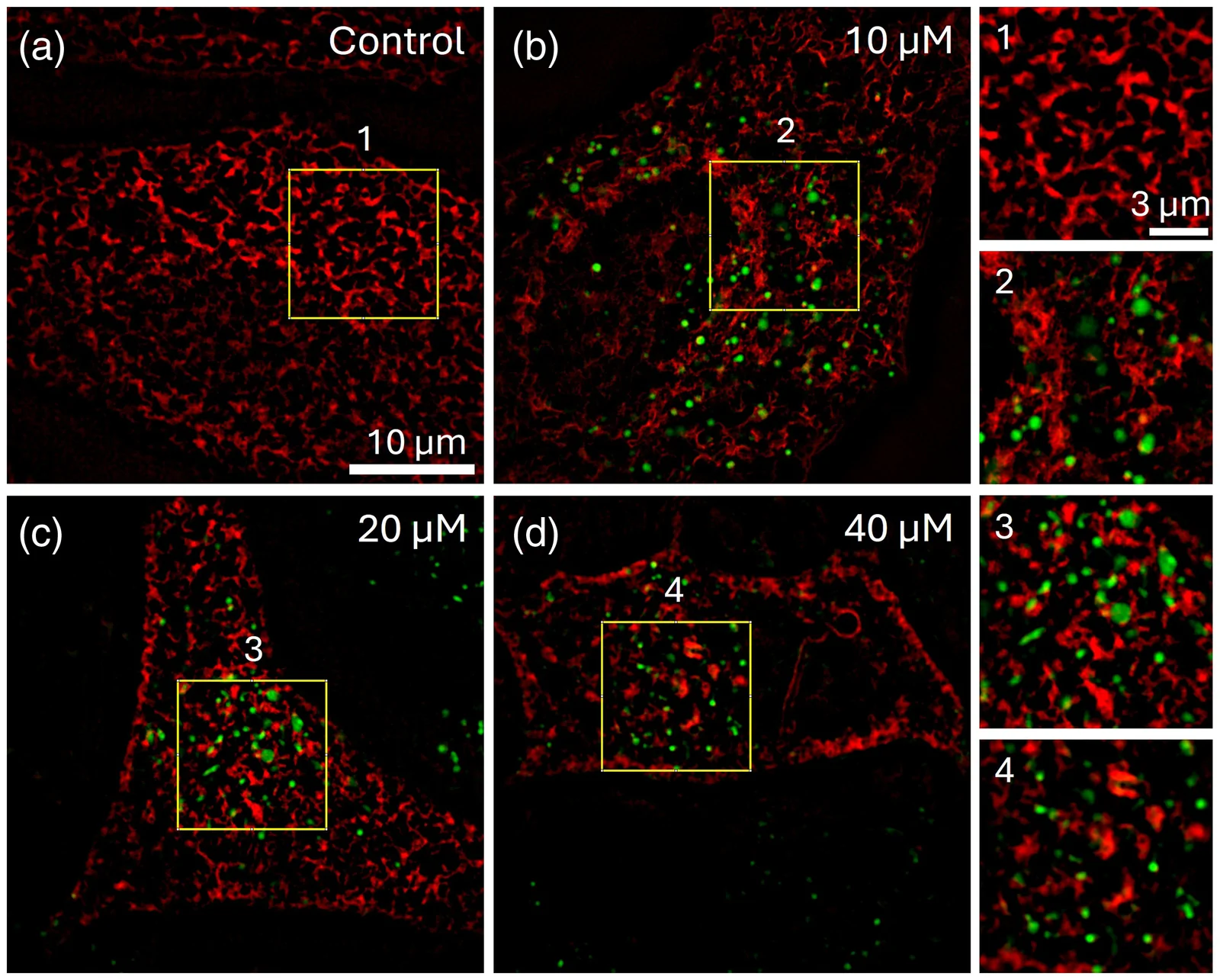
Effect of sunitinib on ER morphology in HeLa cells. (a) SIM image of untreated HeLa cells showing a well-organized, reticular ER network distributed throughout the cytoplasm. (b)–(d) SIM images of cells treated with sunitinib at 10, 20, and 40  μM, respectively, displaying ER (red) and sunitinib fluorescence (green). Exposure to increasing drug concentrations causes progressive disruption of ER architecture, transitioning from an extended interconnected network to fragmented and disjointed structures. Zoomed-in regions of interest, outlined by yellow boxes in panels (1)–(4), provide a high-resolution visualization of ER deformation at different drug concentrations.

**Fig. 4:**
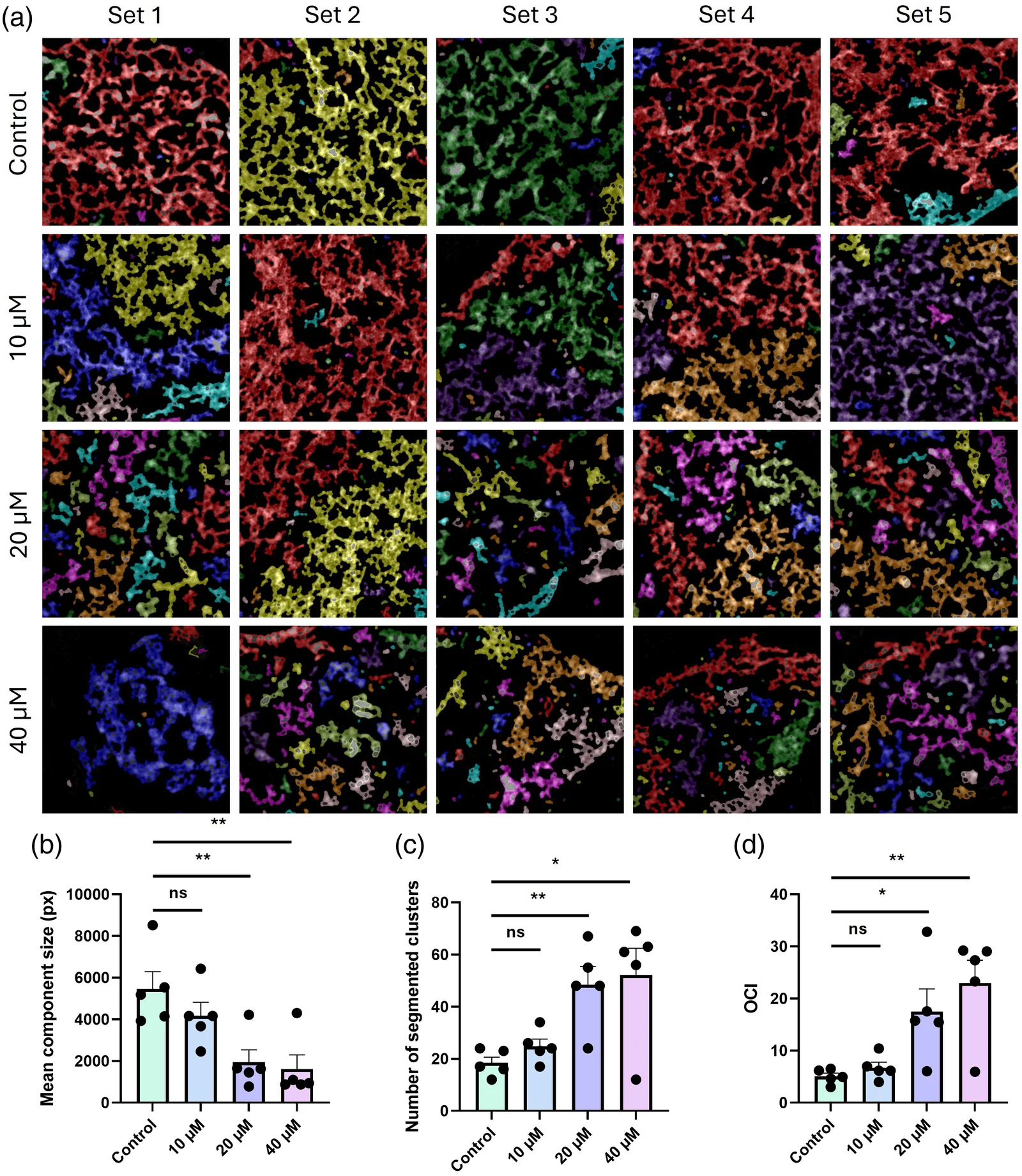
Quantitative topological analysis of ER fragmentation induced by sunitinib. (a) Topological overlay of ER networks generated by assigning pseudo-colors to each independently connected ER segment, with each distinct color representing a spatially disconnected component. Increasing sunitinib concentration results in pronounced ER disorganization and fragmentation, as indicated by higher color multiplicity. (b) Analysis of mean ER component size following sunitinib treatment. (c) Quantification of the number of independent ER components at different drug concentrations. (d) Calculation of the OCI showing progressive increase with sunitinib dosage, indicative of concentration-dependent topological fragmentation of the ER network. Statistical comparisons were performed using an unpaired t test assuming equal variance between groups; p<0.05 was considered statistically significant. *p<0.05, **p<0.01, ***p<0.001.

We further examined whether sunitinib-induced stress extended to the ER, a key organelle responsible for protein folding and calcium homeostasis.^34^^–^^36^ HeLa cells expressing mCherry-ER-3 were treated with increasing concentrations of sunitinib (10 to 40  μM) for 24 h and imaged using SIM. Under control conditions, the ER exhibited a continuous, reticular network distributed throughout the cytoplasm. By contrast, sunitinib treatment induced a concentration-dependent disruption of this architecture, resulting in fragmented and disorganized ER structures (Figs. 3(a)–3(d)]. Such structural disintegration is indicative of ER stress and may contribute to the dysregulation of calcium homeostasis, thereby facilitating ER stress-mediated signaling and pathogenic cross-talk with mitochondria. To comprehensively quantify this disorganization, topological overlays were generated by assigning pseudo-colors to each independently connected ER segment [Figs. 4(a) and S5 in Supplementary Material). In control cells, large, uniform color domains reflected high network connectivity, whereas sunitinib-treated cells exhibited increasingly heterogeneous patterns, indicating extensive fragmentation. Quantitative analysis revealed a reduction in the mean component size [Fig. 4(b)] and a concomitant increase in the number of independent ER fragments [Fig. 4(c)]. To further formalize this observation, we calculated the organelle connectivity index (OCI),^37^ defined as the ratio of the total number of distinct ER fragments to their cumulative network area ($\mu m^{2}$). OCI values increased steadily with drug concentration, confirming a dose-dependent loss of ER connectivity.

Together, these findings indicate that sunitinib exerts a broad, coordinated disruptive effect on key intracellular organelles, including mitochondria, lysosomes, and the ER, ultimately leading to structural fragmentation, impaired function, and loss of cellular viability. The concentration-dependent deterioration observed across these compartments suggests that sunitinib induces an integrated organellar stress response that converges on the collapse of cellular homeostasis. Although our data convincingly demonstrates lysosomal accumulation of sunitinib, as supported by the stable Pearson correlation coefficient, the effects on mitochondrial hyperfusion disruption and ER fragmentation remain correlative. One plausible mechanism is that lysosomal stress perturbs calcium homeostasis, thereby promoting DRP1 activation and mitochondrial fission while also affecting ER architecture through inter-organelle crosstalk.^38^^–^^40^ In addition, disruption of lysosome-mitochondria contact sites may further amplify these stress responses, consistent with prior evidence that tyrosine kinase inhibitors can reprogram organelle communication and induce coupled lysosomal, mitochondrial, and ER dysfunction.

### 2.1 Conclusion

In summary, our findings reveal that sunitinib exerts a comprehensive, concentration-dependent cytotoxic effect on HeLa cells through the simultaneous disruption of multiple intracellular organelles. Beyond its well-established role as a tyrosine kinase inhibitor, sunitinib displays intrinsic fluorescence, allowing real-time visualization of its intracellular distribution. Our imaging analyses demonstrate that the drug preferentially localizes within lysosomes, where it induces destabilization of membrane integrity and morphological deformation. Concurrently, sunitinib treatment provokes severe mitochondrial fragmentation and ER network collapse, both of which are hallmark indicators of cellular stress and metabolic dysfunction. The observed loss of organelle connectivity, manifested by mitochondrial hyperfusion loss, lysosomal depletion, and ER fragmentation, points to a coordinated organellar stress response that undermines cellular architecture and viability. Collectively, these results suggest that the cytotoxicity of sunitinib is mediated, not only by its enzyme-targeting capacity, but also by its physical interactions with cellular membranes and its ability to trigger lysosomal, mitochondrial, and ER dysfunction. This multi-organelle impairment underscores a previously unrecognized mechanism of sunitinib action while also providing a framework for re-evaluating its intracellular pharmacodynamics and toxicity in both cancer and noncancer contexts.

Finally, we would like to emphasize that this study highlights the value of super-resolution live-cell imaging as a powerful approach for resolving the subcellular dynamics of drug molecules in real time. The ability to visualize sunitinib localization and its organelle-specific effects beyond the diffraction limit was essential for uncovering its multi-organelle mode of action. Building on this, future integration of super-resolution imaging with high-throughput and high-content platforms could enable systematic analysis of drug behavior across diverse cellular contexts. Such developments would extend the type of mechanistic insights demonstrated here toward larger-scale, quantitative investigations, ultimately supporting a more comprehensive understanding of intracellular pharmacodynamics. In addition, coupling these imaging strategies with complementary functional assays and computational analysis may further refine our ability to link spatial drug distribution with downstream cellular responses. Together, these advances could help bridge the gap between detailed single-cell observations and predictive, systems-level models of drug action in living cells.

## Supplementary Material

10.1117/1.BIOS.3.4.043102.s01

## Data Availability

The data that supports the findings of this study are available from the corresponding author upon reasonable request.
