# Supplementary material for: Sunitinib induces coordinated mitochondrial, lysosomal, and endoplasmic reticulum disruption, leading to cellular collapse
Source: Biophotonics Discov. 2026 Aug 19;3(4):043102. doi: 10.1117/1.BIOS.3.4.043102 (PMC13557452; doi:10.1117/1.BIOS.3.4.043102)
Supplement: Supplementary file 1 [file BIOS_003_043102_SD001.pdf]

Sunitinib induces coordinated mitochondrial, lysosomal, and endoplasmic reticulum disruption, leading to cellular collapse

Aditya Yadav,[1] Eugene Lee,[1] Landen Liu,[1] and Jiajie Diao[1]*
[1]Department of Cancer Biology, University of Cincinnati College of Medicine, Cincinnati, OH 45267, USA.
**Corresponding author: jiajie.diao@uc.edu (J.D.)*

Experimental

Materials

Sunitinib (Cat. No. HY-10255A) was purchased from MedChemExpress. LysoTracker Red DND-99 (No. L7528, LTR) and MitoTracker Deep Red FM (No. M22426, MTDR) were purchased from Invitrogen (Thermo Fisher Scientific, USA). Dulbecco's modified Eagle's medium (No. 11965092, DMEM), fetal bovine serum (No. 26140079, FBS), and penicillin-streptomycin (10,000 units/mL, No. 15140163) were purchased from Gibco (Thermo Fisher Scientific, USA). Phosphate-buffered saline (No. SH30256.01, PBS) was purchased from Hyclone (GE Healthcare Life Sciences, USA).

Preparation of Sunitinib solution and cell culture

The desired amount of MXenes was dissolved in DMSO to prepare a stock solution (10 mM) before the cell experiments. The solution was diluted in the DMEM with 10% FBS for the treatment of cells. DMEM with penicillin-streptomycin (100 units/mL) and FBS (10%) was used to culture the cells. Cells were grown in an incubator (Thermo Fisher Scientific, USA; 37 °C, 100% humidity, 5% CO_2).

Mitochondria, endoplasmic reticulum (ER), and lysosomes labeling

The grown cells were split and cultured in a glass-bottomed micro-well dish and incubated with 2 mL of DMEM supplemented with 10% FBS for 24 h. For transfection with mCherry-ER-3, lysosome, and mitochondria staining, we followed the procedure as mentioned by the supplier.

Structured illumination microscopy imaging

A Nikon structured illumination microscopy with three 3D-SIM channels (N-SIM, Tokyo, Japan) was used to perform all SIM images. A NIS-Elements AR Analysis was used to reconstruct all the raw images. For Sunitinib imaging, an emission bandwidth at 500-550 nm and a 488 nm laser were used. For LTR imaging, an emission bandwidth at 570-640 nm and a 561 nm laser were used. For MTDR imaging, an emission bandwidth at 660-735 nm and a 640 nm laser were used.

Cell viability assay

Cells were treated in a 96-well plate at a density of 5×10^5 cells/mm. The viability was determined by using a cell counting kit-8 (CCK-8). 10 µL CCK-8 solution was added to each well, and the OD value for each well was read at a wavelength of 450 nm on a microplate reader (Multiskan, Thermo, USA).

ER analysis

An automated image analysis pipeline was developed to quantify network topology from the acquired micrographs. Multi-channel images were first converted to a single-channel grayscale representation and subjected to min-max normalization to standard 8-bit intensities (0-255) for consistent contrast. Structural boundaries were then extracted using the Canny edge detection algorithm, employing a Gaussian filter with a standard deviation of 1.0 to mitigate high-frequency noise prior to spatial gradient computation and hysteresis thresholding. To reconstruct continuous structures from the locally discontinuous edge maps, a

sequence of mathematical morphological operations utilizing disk-shaped structuring elements was applied: a dilation operation (radius = 3 pixels) bridged narrow spatial gaps, an erosion operation (radius = 2 pixels) refined the artificially thickened boundaries back toward their true physical dimensions, and a morphological closing (radius = 3 pixels) filled small internal voids and fused narrow breaks. Spurious background artifacts were subsequently eliminated by filtering out any isolated contiguous objects comprising fewer than 40 pixels. The refined binary masks then underwent connected-component labeling based on orthogonal spatial connectivity to isolate and iteratively count independent, completely disconnected network fragments. Finally, absolute quantitative topometric calculations were performed utilizing a predefined spatial resolution of 0.00625 μm/pixel.

Mathematical framework for topology analysis

The automated topometric analysis consists of a sequential mathematical pipeline designed to extract, reconstruct, and quantify the spatial network topology from raw two-dimensional micrographs. The step-by-step formulations governing this process are detailed below.

1. Image preprocessing and normalization

Let the raw grayscale input image be defined as a discrete spatial matrix $I(x, y)$, where (x, y) represents the pixel coordinates. To standardize pixel intensities across diverse imaging conditions, a min-max normalization is applied to map the intensity values to a standard 8-bit range [0,255]:

$$I_{\text{norm}}(x, y) = \frac{I(x, y) - \min(I)}{\max(I) - \min(I)} \times 255$$

2. Canny edge detection

To isolate the structural boundaries, the normalized image is convolved with a two-dimensional Gaussian kernel $G(x, y)$ with a standard deviation of $\sigma = 1.0$ to suppress high-frequency noise:

$$I_{\text{smooth}}(x, y) = I_{\text{norm}}(x, y) * G(x, y)$$

where

$$G(x, y) = \frac{1}{2\pi\sigma^2} e^{-\frac{x^2+y^2}{2\sigma^2}}$$

The spatial gradient magnitude $|\nabla I|$ and direction θ are then computed using discrete spatial derivatives:

$$|\nabla I| = \sqrt{\left(\frac{\partial I_{\text{smooth}}}{\partial x}\right)^2 + \left(\frac{\partial I_{\text{smooth}}}{\partial y}\right)^2}$$

$$\theta = \arctan\left(\frac{\partial I_{\text{smooth}}/\partial y}{\partial I_{\text{smooth}}/\partial x}\right)$$

Following non-maximum suppression along the gradient direction θ and subsequent hysteresis thresholding, a binary edge map $E(x, y)$ is generated, where $E(x, y) \in \{0,1\}$.

3. Mathematical morphology

To bridge gaps and reconstruct continuous structural features from the extracted edge map, a sequence of morphological operations is applied using a circular (disk-shaped) structuring element B_r, where r denotes the pixel radius.

Dilation

The edge map is first dilated using $r = 3$ to connect proximal fragments. This is defined by the Minkowski addition:

$$D(x,y) = (E \oplus B_3)(x,y) = \max_{(i,j)\in B_3} E(x-i, y-j)$$

Erosion

To refine the artificially thickened boundaries while preserving the newly formed bridges, an erosion with $r = 2$ is applied:

$$R(x,y) = (D \ominus B_2)(x,y) = \min_{(i,j)\in B_2} D(x+i, y+j)$$

Closing

Finally, a morphological closing operation (a dilation followed by an erosion) with $r = 3$ is utilized to fill small internal voids and consolidate the topology:

$$C(x,y) = (R \cdot B_3)(x,y) = (R \oplus B_3) \ominus B_3$$

4. Spatial filtering and connected component analysis

To eliminate spurious background noise, an area-opening filter is applied to the closed binary image $C(x,y)$. Let the image be partitioned into a set of k disjoint connected components $\{S_1, S_2, \dots, S_k\}$ based on 4-way orthogonal pixel connectivity. The filtered binary mask $M(x,y)$ is formed by discarding any component S_i with a pixel area $|S_i|$ strictly less than the threshold $\tau = 40$ pixels:

$$M(x,y) = \bigcup \{S_i \mid |S_i| \geq 40\}$$

The remaining valid components in $M(x,y)$ are subsequently assigned unique numerical identifiers. Let N denote the total number of surviving, distinct connected components.

5. Topometric quantification

Absolute spatial metrics are derived by scaling the segmented pixel data to physical dimensions. Let the predefined physical pixel resolution be denoted as $p = 0.00625$.

The total physical segmented area A_{total} (in μm^2) is calculated as the sum of all pixels constituting the N components multiplied by the square of the pixel resolution:

$$A_{\text{total}} = \left(\sum_{i=1}^{N} |S_i|\right) \times p^2$$

To quantify network fragmentation, the Organelle Complexity Index (OCI) is defined as the spatial density of discrete, disconnected structures relative to the total segmented area:

$$OCI = \frac{N}{A_{\text{total}}}$$

Additionally, the mean cross-sectional area of the individual network components $\bar{A}_{\text{px}}$ (expressed in pixels) is determined by computing the arithmetic mean of the isolated object sizes:

$$\bar{A}_{\text{px}} = \frac{1}{N}\sum_{i=1}^{N} |S_i|$$

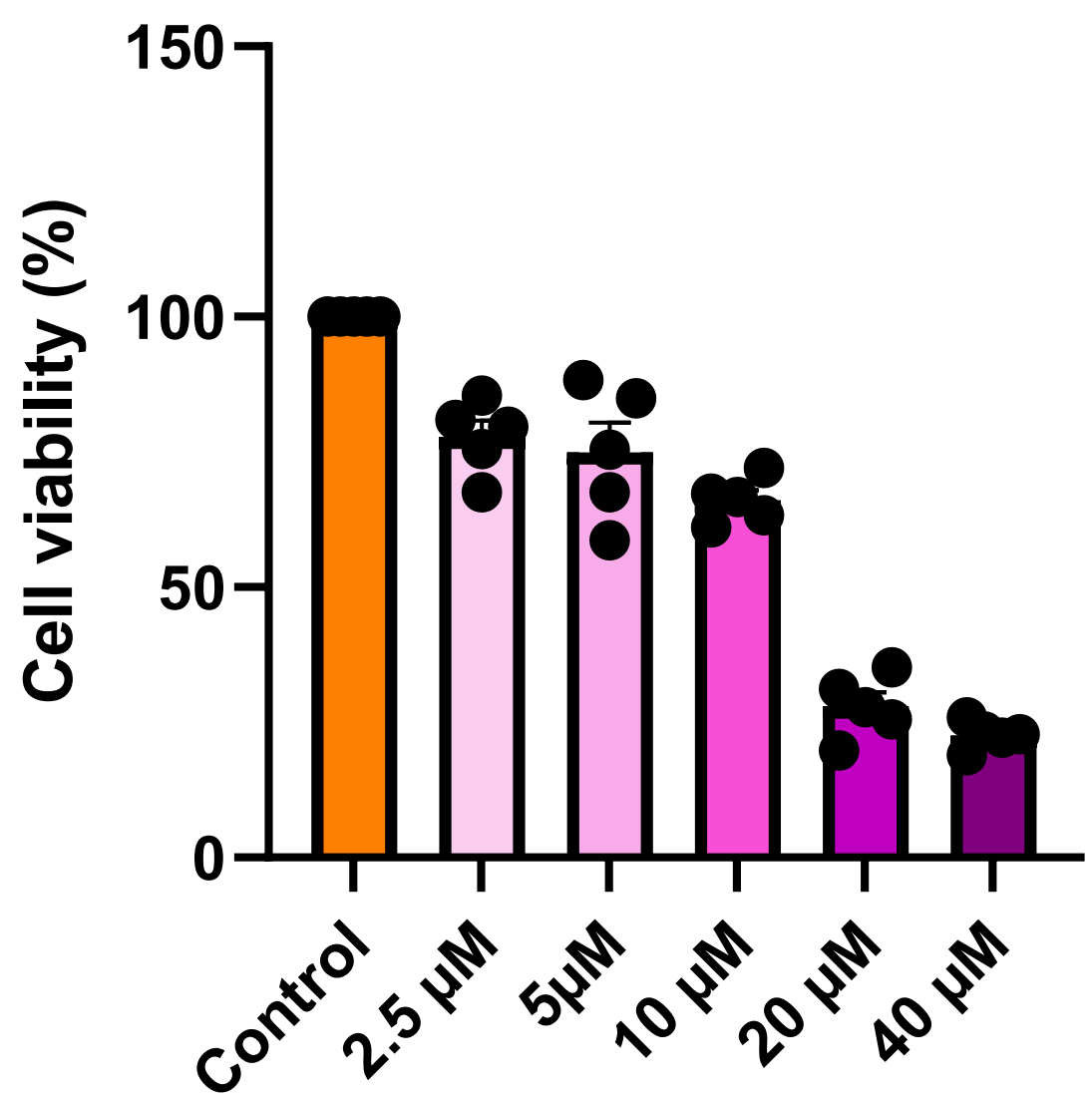

Figure S1. CCK-8 assay for assessment of sunitinib cytotoxicity in HeLa cells. Cells were treated with sunitinib for 12 h. Data are presented as mean ± SEM.

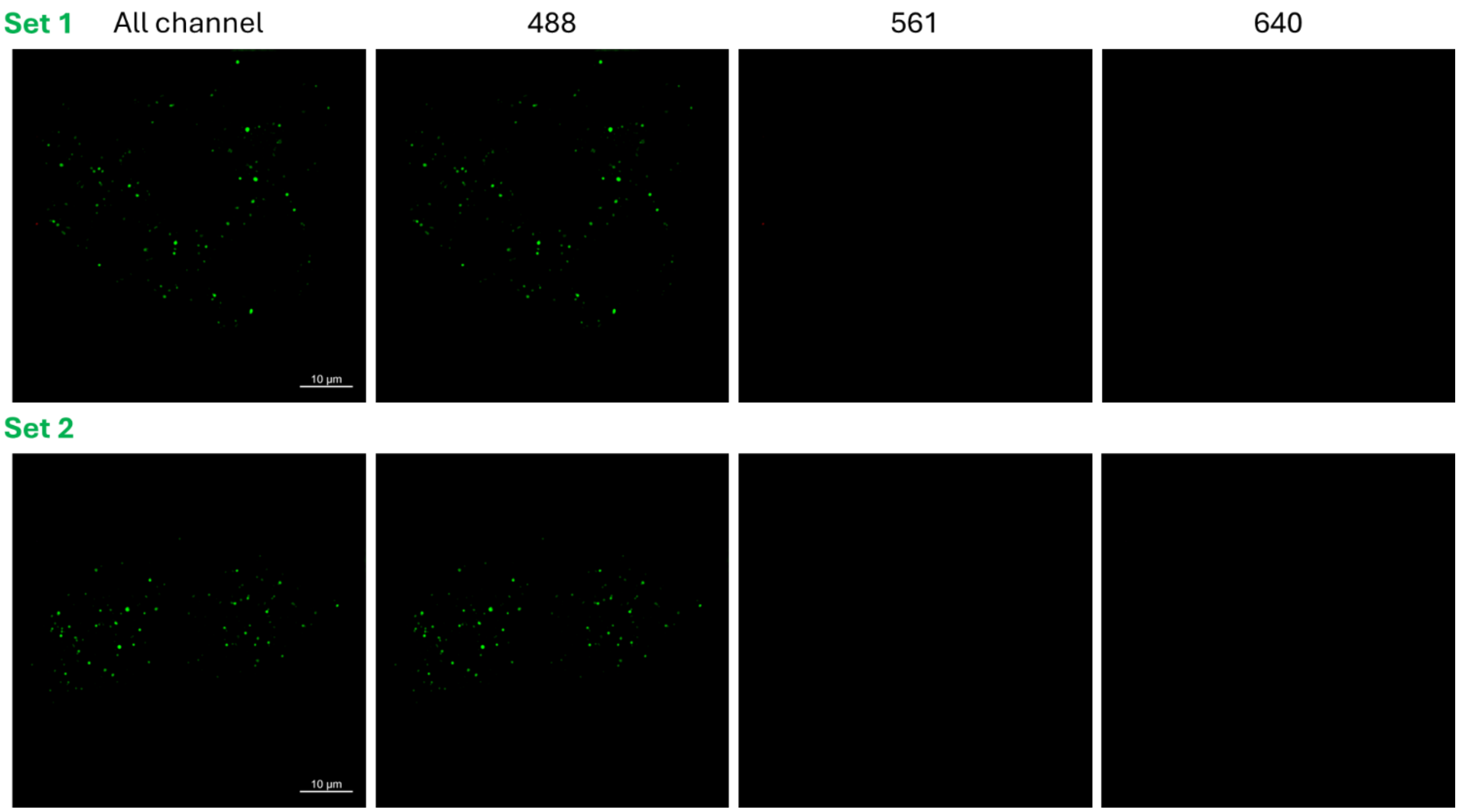

Figure S2. SIM images of sunitinib under different excitation wavelengths.

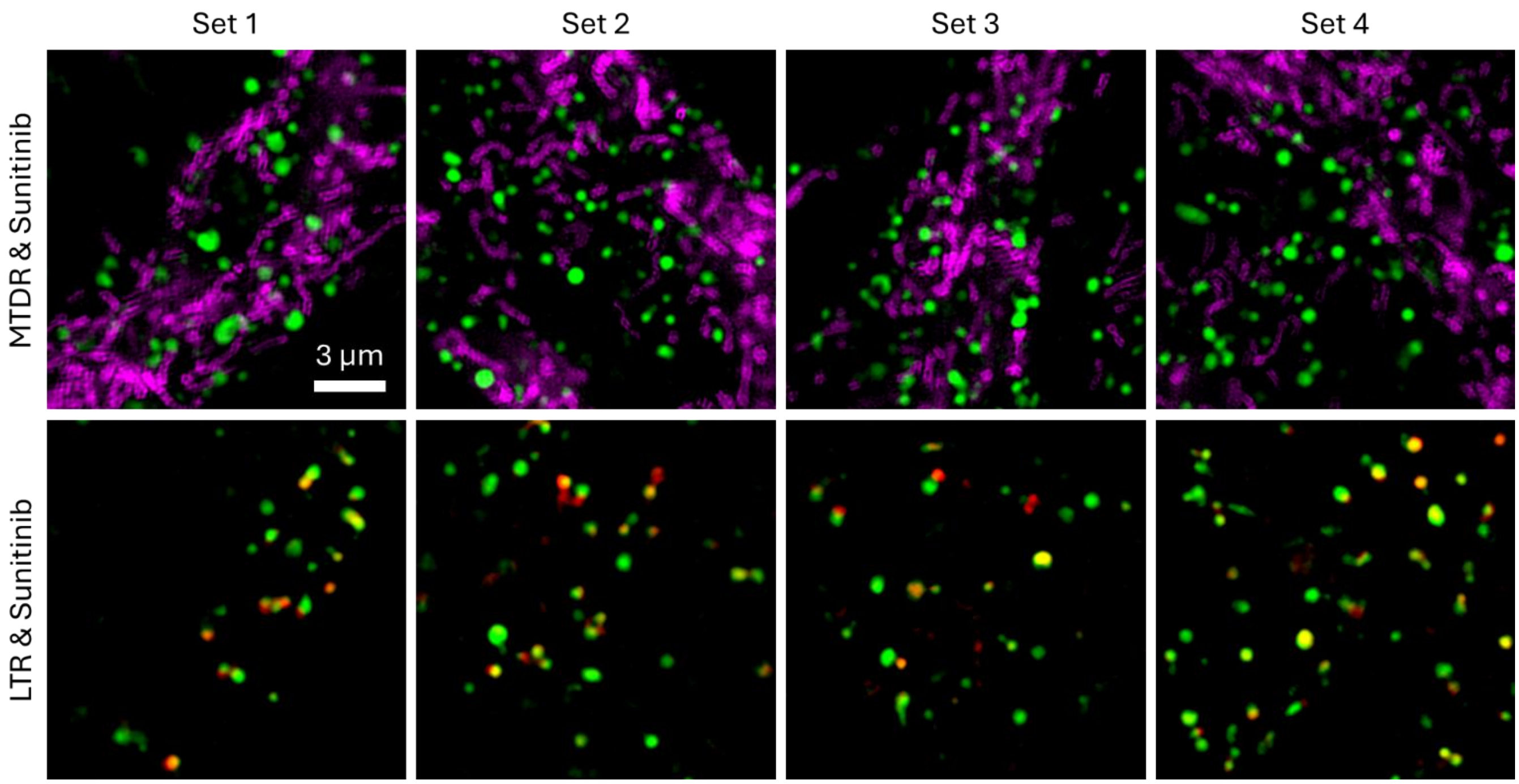

Figure S3. Organelle-specific localization of sunitinib in HeLa cells. HeLa cells were co-stained with MitoTracker Deep Red (MTDR, magenta) and sunitinib (green) in one set, and with LysoTracker Red (LTR, red) and sunitinib (green) in a separate set.

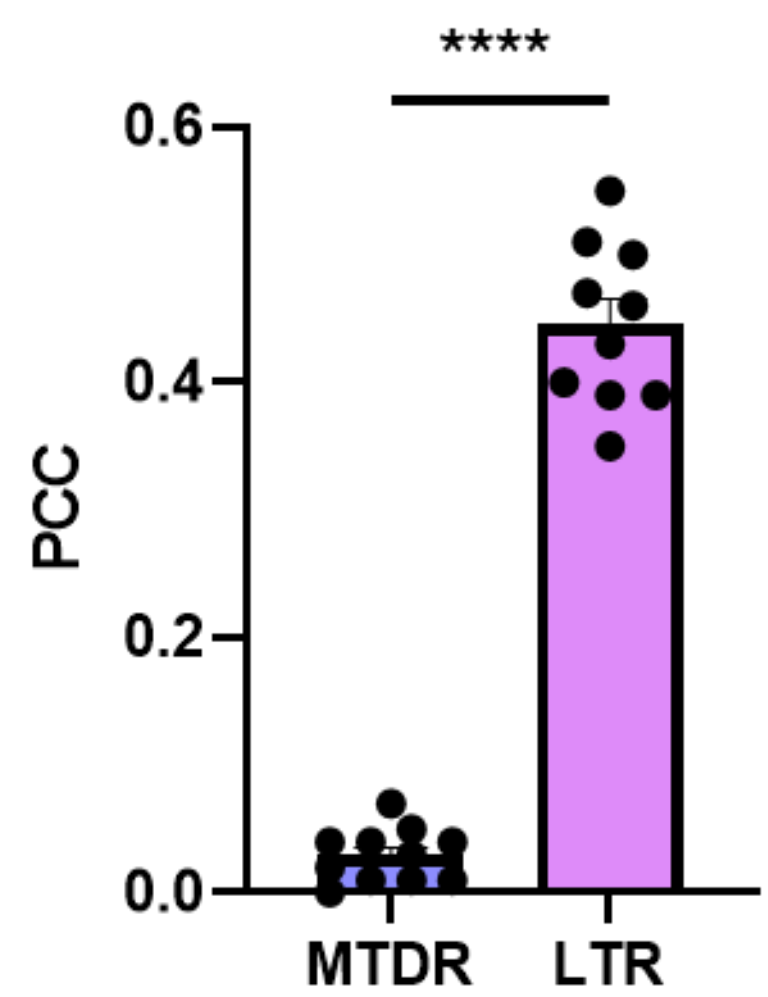

Figure S4. Pearson's correlation coefficient (PCC) analysis between the Sunitinib channel and MTDR, and between the Sunitinib channel and LTR, respectively.

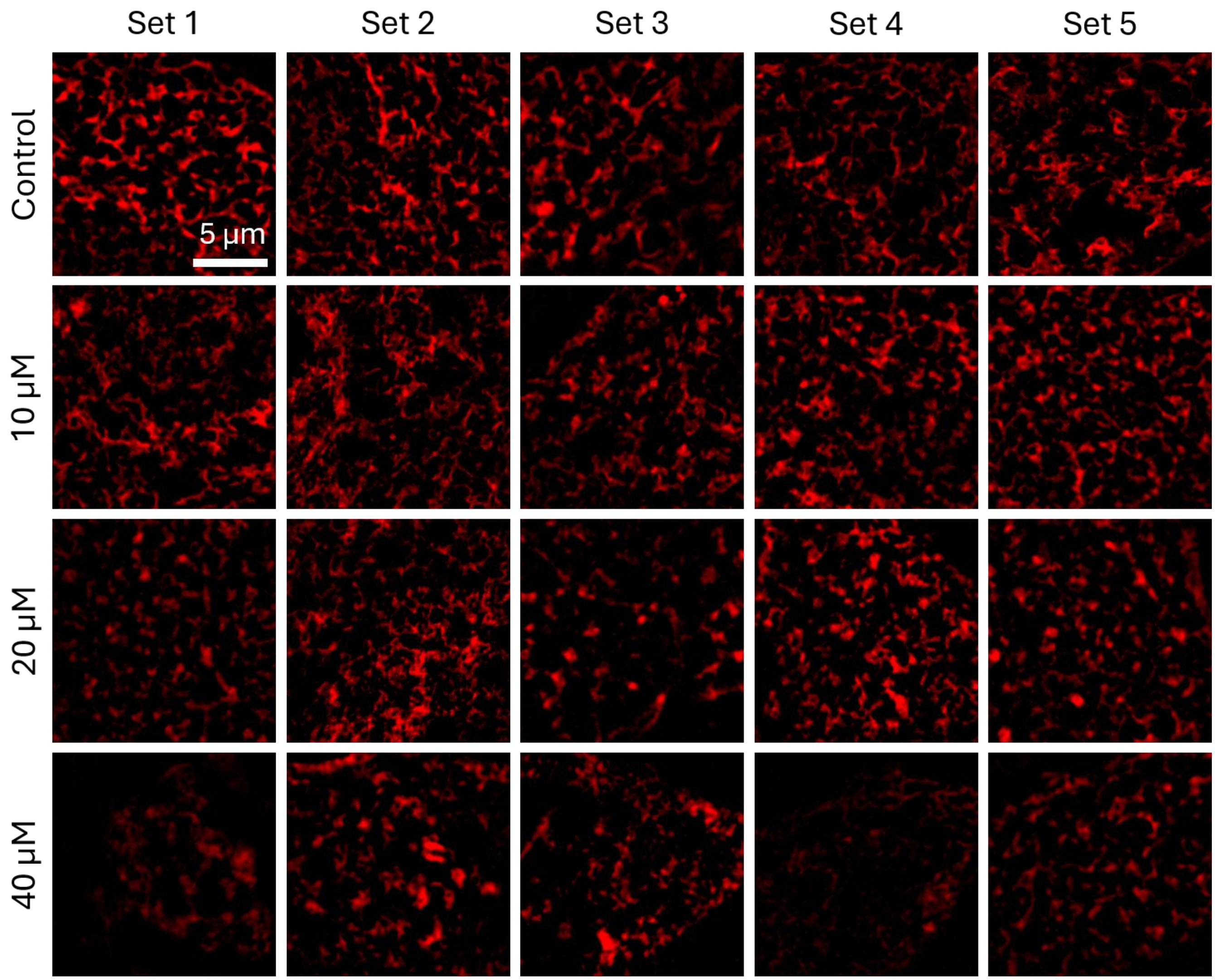

Figure S5. Data set used for ER analysis.
